# In Vitro Analysis of Extracts of Plant Used in Mexican Traditional Medicine, Which Are Useful to Combat *Clostridioides difficile* Infection

**DOI:** 10.3390/pathogens11070774

**Published:** 2022-07-07

**Authors:** Jacqueline E. Martínez-Alva, Emilio Espinoza-Simón, Yuli Bayona-Pérez, Nancy C. Ruiz-Pérez, Sara A. Ochoa, Juan Xicohtencatl-Cortes, Javier Torres, Mariana Romo-Castillo

**Affiliations:** 1 Campus Chapultepec, Universidad del Valle de México, Mexico City 11810, Mexico; jacqui_971208@hotmail.com (J.E.M.-A.); emilio.espinoza.simon@gmail.com (E.E.-S.); 2 División Académica de Ciencias de la Salud, Universidad Juárez Autónoma de Tabasco, Villahermosa 80040, Mexico; yuli_ewi_bayona@hotmail.com (Y.B.-P.); nancy_90_116@hotmail.com (N.C.R.-P.); 3 Laboratorio de Investigación en Bacteriología Intestinal, Hospital Infantil de México “Federico Gómez”, Mexico City 06720, Mexico; saraariadnah@hotmail.com; 4 Unidad de Investigación Médica en Enfermedades Infecciosas y Parasitarias, Hospital de Pediatría, Centro Médico Nacional Siglo XXI, IMSS, Mexico City 06720, Mexico; 5 Cátedras de Consejo Nacional de Ciencia y Tecnología (CONACYT), Posgrado en Fitosanidad-Fitopatología, Colegio de Postgraduados, Texcoco 56230, Mexico

**Keywords:** natural extract, antimicrobial activity, Mexican traditional medicine, *Clostridioides difficile*, action mechanism, clinical strains, antibiotic resistance

## Abstract

Recently, a worrying acceleration of the emergence of antibiotic-resistant bacteria has been reported. The increase in antibiotic-associated diseases, such as *Clostridioides difficile* infection (CDI), has promoted research on new treatments that could be more effective and less aggressive for CDI patients. This study evaluates eight plants with antimicrobial activity commonly used in Mexican traditional medicine to evaluate their potential against *C. difficile*. We provide essential information about these plants’ activities and action mechanisms against *C. difficile* and their effect on different bacterial infection activities: motility, adherence, sporulation, and germination. The selected plants are rosemary, estafiate, rue, epazote, mint, toloache, ajenjo, and thyme. We used clinical isolates to test their activity against strains responsible for current outbreaks to provide more information about the clinical impact of these extracts. We found that thyme, ajenjo, and mint were the most effective against the isolates. We identified that the extracts affected protein synthesis. In addition, the extracts affect the strains’ motility, and some, such as thyme extract, affect adherence, whereas rue extract affects sporulation. These results led to the identification of new compounds beneficial to CDI treatment.

## 1. Introduction

Antibiotic resistance is a global health problem, and the principal cause has been the indiscriminate use of antibiotics. Between 2000 and 2010, the consumption of antibiotics in the world increased by 70% [1]. The World Health Organization (WHO) estimated that two tons of antibiotics are used every 10 min, most without a medical prescription. *Clostridioides difficile* is considered the primary cause of antibiotic-associated diarrhea. *C. difficile* infection (CDI) therapy includes metronidazole and vancomycin as the first choice. Recently, rifaximin, ridinilazole, and new antibiotics have been proposed as additional therapies [2]. Unfortunately, resistant *C. difficile* strains are increasing, making this infection challenging to eradicate.

In Mexico, pre-Hispanic cultures developed many uses for medicinal plants, many of which are still traditionally used today. Additionally, historical events such as the Spanish conquest influenced the use of Mexican plants and the introduction of new species to the Mexican flora that persist in traditional medicine [3]. However, although Mexico is rich in medicinal plants, their use is poorly studied. Traditional Mexican medicine uses more than 3000 plants to treat various diseases, ranking second worldwide [4]. Herbal culture is transmitted orally from generation to generation, and in many places, it is preferred over prescriptions because of its lower cost or natural origin. The plant families with the most sizable number of species with antibacterial activity are Asteraceae (16%), Fabaceae (8%), and Lamiaceae (6%) [5,6]. *Artemisia ludoviciana*, *Artemisia absinthium*, and *Calendula officinalis* are in the Asteraceae family. *Acacia cornigera*, *Caesalpinia pulcherrima*, *Haematoxylum brasiletto*, *Mimosa pigra*, and *Senna skinneri* are in the Fabaceae family. *Mentha piperita*, *Origanum vulgare*, *Rosmarinus officinalis*, and *Thymus vulgaris* species with antibacterial activity in the Lamiaceae family [5].

Previous studies demonstrated that some condiments, such as garlic, cinnamon, ginger, and turmeric, have a significant effect against *C. difficile*. Additionally, onion and coconut oil affect sporulation. The primary action mechanism of the extracts previously analyzed is membrane damage [7,8,9,10]. This study’s main objective was to examine the antibacterial effect of various plant extracts against *C. difficile* clinical isolates in traditional Mexican medicine. Elucidation of their impact on clinical strains can help identify new antibacterial therapies. We also analyzed the effect of the extracts on some virulence factors. Finally, we tried to elucidate their action mechanism to determine the best candidate for deeper future analysis.

## 2. Results

### 2.1. Phytochemical Analysis

First, we decided to perform a preliminary phytochemical analysis to determine the presence of secondary metabolites. Usually, the composition of plant extracts is subject to changes under the influence of several factors, such as maturity stage, widespread use of pesticides, storage conditions, environmental conditions, and genomic composition. This variation could result in different profiles and activities. The phytochemical composition of our extracts varied considerably (Table 1), leading us to the possibility of identifying new plant extracts that act against CDI. The mint extract contained only polyphenols; rue contains alkaloids, sterols, terpenoids, and polyphenols. Because this study aimed to identify complete plants that could be useful for therapies against CSI, we want only to determine which type of secondary metabolites were present.

### 2.2. Antibacterial Activity Assay and Minimal Inhibitory Concentration (MIC) Determination

**Figure 1:**
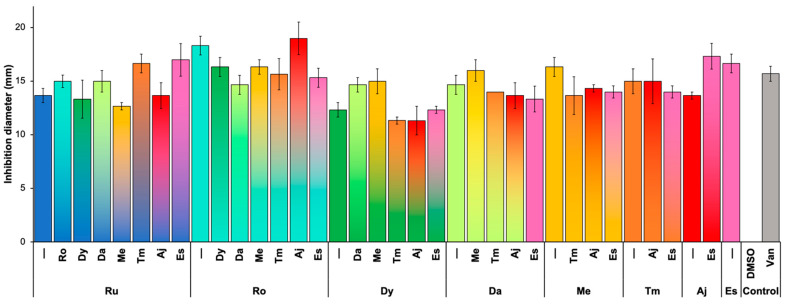
Inhibition effect and synergistic antimicrobial activity of plant extracts against *C. difficile*. Extract nomenclature: rue (Ru), rosemary (Ro), epazote (Dy), toloache (Da), mint (Me), thyme (Tm), ajenjo (Aj), and estafiate (Es). Dimethyl sulfoxide (DMSO) and vancomycin (Van) were used as controls. Colors represent the mixtures of the extracts: Ru—blue, Ro—cyan, Dy—green, Da—lime, Me—yellow, Tm—orange, Aj—red, Es—pink, DMSO—purple, and Van—gray. The concentrations of the extracts were the MICs reported in Table 1.

Evaluation of the antibacterial activity of our plant extracts by the disk diffusion method was performed, and the MIC was determined for each one. The MICs shown in Table 1 were used in all the following analyses. The antibacterial activity is shown in Figure 1. We designated a beneficial effect against the strains if the inhibition ratio was ≥15 mm [11]. The results revealed that rosemary, mint, and estafiate had a significant inhibitory effect comparable with vancomycin.

### 2.3. Synergism between Plant Extracts

Previous studies have reported that combinations of plant extracts could potentiate their antibacterial activities [12,13]. Therefore, to evaluate the possible synergistic effect between our extracts, determination of growth inhibition by disk diffusion assay was performed using a 1:1 mixture of extracts. Unfortunately, no significant synergistic effect was found between our extracts (Figure 1).

### 2.4. Action Mechanism of Plant Extracts

**Figure 2:**
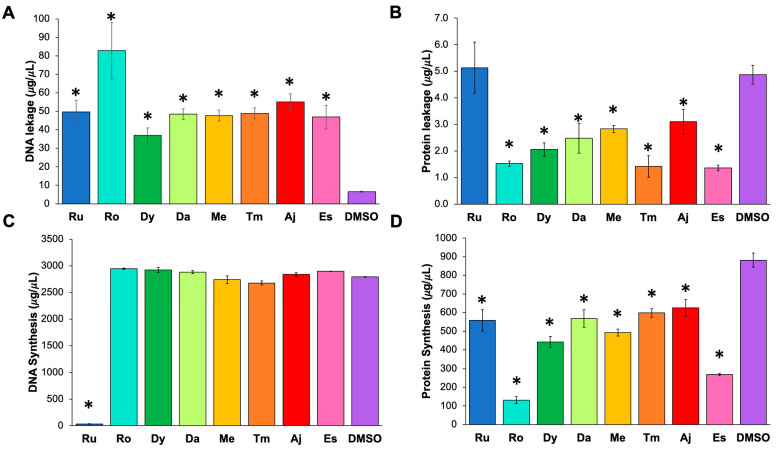
Action mechanism of plant extracts against *C. difficile*. (**A**) DNA leakage. (**B**) Protein leakage. (**C**) DNA synthesis. (**D**) Protein synthesis. Extract nomenclature: rue (Ru, blue), rosemary (Ro, cyan), epazote (Dy, green), toloache (Da, lime), mint (Me, yellow), thyme (Tm, orange), ajenjo (Aj, red), and estafiate (Es, pink). Dimethyl sulfoxide (DMSO, purple) was used as a control. The concentrations of the extracts were the MICs reported in Table 1. Statistical differences were determined using one-way ANOVA plus the Bonferroni and Holm comparison test: * *p* < 0.05.

To better understand the mechanism or target of the extracts against the bacteria, we implemented a method to identify the possible route of action. The technique selected allowed us to determine if the extract affects cell membrane permeability or impairs nucleic acid or protein synthesis. The results showed more DNA in the supernatant of bacteria exposed to the rosemary extract and a higher protein quantity in the supernatant of bacteria exposed to the rue extract. These results suggest that rosemary induces DNA leakage (Figure 2A), whereas rue causes protein leakage compared with the other extracts (Figure 2B). The protein leakage effect observed by DMSO was not understood; we suggest that it could be an intrinsic effect of DMSO that plant extracts could mask, but more analysis is needed to conclude that. When we analyzed cytoplasmic DNA and protein concentrations, only a significant decrease in DNA concentration was shown in bacteria treated with the rue extract.

In contrast, the protein concentration in the cytoplasm of bacteria treated with all extracts decreased significantly in bacteria treated with the estafiate and rosemary extracts. These results suggest that the rue extract was the only one that affected DNA synthesis (Figure 2C). Although all the extracts affected protein synthesis, those of rosemary and estafiate had the most significant impact (Figure 2D). The action of some of our extracts could not be identified in our analysis, suggesting that other mechanisms not analyzed in this study, such as impaired respiration, metabolism, or ATP production, could be involved [14].

### 2.5. Effect of the Extracts on Virulence Factors

**Figure 3:**
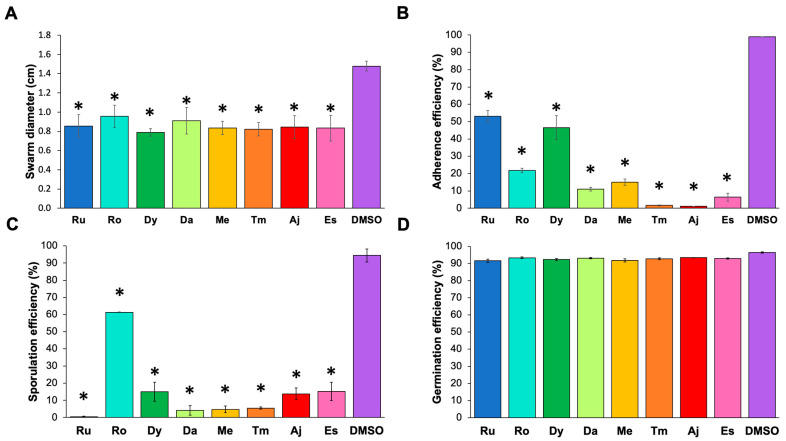
Effect of plant extracts against *C. difficile* virulence factors. (**A**) *C. difficile* motility (swarming). (**B**) *C. difficile* adhesion to HT-29 cells. (**C**) Sporulation efficiency of *C. difficile*. (**D**) Germination efficiency of *C. difficile*. Extract nomenclature: rue (Ru, blue), rosemary (Ro, cyan), epazote (Dy, green), toloache (Da, lime), mint (Me, yellow), thyme (Tm, orange), ajenjo (Aj, red), and estafiate (Es, pink). Dimethyl sulfoxide (DMSO, purple) was used as a control. The concentrations of the extracts were the MICs reported in Table 1. Statistical differences were determined using one-way ANOVA plus the Bonferroni and Holm comparison test: * *p* < 0.05.

We analyzed the effects of the extracts on virulence activities such as motility, adherence, sporulation, and germination in *C. difficile*. These factors play a principal role in CDI. The analyses revealed that all the extracts significantly affected strain motility, reducing it by 40% (Figure 3A). In addition, although all the extracts eliminated the adhesion of the bacteria to HT-29 cells, thyme, ajenjo, and estafiate had a significant inhibitory effect on this process (Figure 3B). None of the extracts affected spore germination (Figure 3D), but rue, toloache, mint, and thyme extracts inhibited *C. difficile* sporulation (Figure 3C).

## 3. Discussion

The identification of new therapies for use against pathogens is a health priority. Unfortunately, similar to CDI, some bacterial infections have no effective treatment, leading to the possibility of reinfections and making their eradication difficult [15]. The current therapy used to control CDI fails to prevent recurrent cases and is expensive. Additionally, identifying new *C. difficile* strains resistant to many antibiotics due to the indiscriminate use of antibiotics has stimulated the search for new treatments or adjuvant therapies against CDI. Many treatments derived from medicinal plants have been proven to be an abundant source of biologically active compounds. Traditional Mexican medicine provides a wide range of valuable medicinal plants to fight various infections. We chose eight of the most common plants to analyze their potential against clinical isolates of *C. difficile*.

The study of the antimicrobial properties of plants has increased in the last few years. Additionally, research on using these plants as efficient antimicrobial drugs against nosocomial diseases and their mechanisms of action is growing. In addition, bioactive compounds provide health benefits. Many studies have analyzed the effects of plant extracts and phytochemicals using standard laboratory strains. However, we decided to explore the effectiveness of the extracts against clinical isolates to show a more relevant result regarding the antimicrobial effect the extracts could have in the clinic.

This study aimed to identify and analyze eight natural products’ mechanism action and effectiveness against *C. difficile* strains. The phytochemical characterization of our plant extracts showed that most have alkaloids, polyphenols, and terpenoids. In the case of mint, our isolation technique allowed us to identify only polyphenols. The concentration of secondary metabolites is associated with cultivation, the harvest season, water availability during irrigation, and soil salinity [16,17]. In general, most plants contain polyphenols, terpenes, and alkaloids. These substances have antimicrobial activity due to the induction of increased membrane permeability [5]. We demonstrated that all the extracts present antimicrobial activity against clinical *C. difficile* strains. We observed that ajenjo and thyme had a relevant inhibitory effect on most strains included. Compared with the antibiotic used as a control (vancomycin), some plant extracts, such as those of rosemary, mint, and estafiate, showed an inhibitory activity close to that observed with the reference antibiotics, suggesting that the components of these extracts could be an alternative therapy against bacterial infections. Although we did not identify a strong synergistic effect against most strains, we propose that a mixture of extracts could be the best choice to enhance their effect and target many virulence factors. However, further studies are needed to confirm this hypothesis.

Members of the Datura family (*Datura stramonium*, *Datura ferox*, etc.) are used for analgesic, anesthetic, and ceremonial purposes [5]. In addition, these plants produce alkaloids known as scopolamine and atropine [18]. However, the secondary effects of these plants’ applications are dangerous; for this reason, the study of the antimicrobial effect of these plants has been limited. However, since pre-Hispanic times, these plants have been used for their anti-inflammatory and antipyretic properties [19]. We identified that toloache has antimicrobial activity against *C. difficile* and plays a valuable role in inhibiting sporulation. Our results are supported by a previous report in which the antimicrobial activity of this plant was demonstrated against *Bacillus subtilis*, *Enterococcus faecalis*, and *Staphylococcus aureus* [20].

Regarding the mechanism of action, we found that some extracts affect membrane permeability, possibly due to the activity of polyphenols and terpenes [21,22,23]. Furthermore, when comparing DNA and protein leakage from the cells after incubation with the extracts, we identified that some extracts induced only DNA or protein leakage (those of rosemary and rue, respectively) but not both. These results suggest that some extracts induce the formation of temporary pores in the membrane, which, depending on their size, selectively allow DNA or protein to leak [24,25]. However, an in-depth analysis is required to support this hypothesis.

We also identified good candidates that could inhibit or affect some virulence factors and the CDI process. Sporulation and Germination processes play a critical role in CDI control since they facilitate the dispersion of the pathogen and protect it from antibiotics. For this reason, in this study, we considered it essential to analyze the potential of plant extracts over these processes. Although all extracts decreased the motility of the *C. difficile* strains, the thyme, ajenjo, and estafiate extracts significantly blocked adherence to HT-29 cells. In contrast, the rue, toloache, mint, and thyme extracts stopped the sporulation process. Previously, some extracts, such as coconut oil and onion extracts, were demonstrated to inhibit *C. difficile* sporulation [4]. Reducing toxin activity and production in *C. difficile* strains were reported, showing that garlic powder, onion bulb, and pomegranate extracts are the best candidates [7,26]. Although none of our extracts affected this virulence activity, we consider that future analyses, including these extracts, must be conducted if we want to find a mixture of compounds that can control all the virulence factors.

Our study confirms the potential of some plant products as antibacterial therapies. Work is in progress to isolate and identify the active compounds, examine the action of extract mixtures, and determine the mechanisms of action against bacteria. The central focus of our laboratory is to find biologically active compounds that can be employed as antibacterial agents, so an initial analysis of global extracts is necessary to select the best candidates to study for therapies against CDI. Additionally, the synergy with antibiotics usually used to combat CDI must be analyzed to identify alternative therapies that enhance the action of those used now to eliminate reinfections.

## 4. Materials and Methods

### 4.1. Plant Extracts

The selected plants were obtained from the famous plant market of Xochimilco in Mexico City. The plants included in this study were rosemary (*Rosmarinus officinalis*, Ro), estafiate (*Artemisia ludoviciana*, Es), rue (*Ruta graveolens*, Ru), epazote (*Dysphania ambrosioides*, Dy), mint (*Mentha piperita*, Me), toloache (*Datura ferox*, Da), ajenjo (*Artemisia absinthium*, Aj) and thyme (*Thymus vulgaris*, Tm). The plants were identified taxonomically using morphological features by Dr. Emilio Espinoza-Simon using the resources availed by the National Commission for the Knowledge and Use of Biodiversity CONABIO (http://www.conabio.gob.mx/malezasdemexico/2inicio/home-malezas-mexico.htm accessed on 31 May 2019), the Missouri Botanical Garden (https://www.missouribotanicalgarden.org/ accessed on 5 June 2019) and the Digital Library of Traditional Mexican Medicine of the National Autonomous University of Mexico (http://www.medicinatradicionalmexicana.unam.mx/ accessed on 8 June 2019). The plants were washed with distilled water and dried in an oven at 40 °C for 48 h. Distinct parts of the selected plants were used (Table 1). First, plants were mashed and immersed in 96% ethanol for 48 h at room temperature. After that, extracts were filtered and concentrated by distillation and resuspended in 10 mL of DMSO to obtain the final concentration (*w*/*v*, Table 1). Finally, the obtained extracts were filtered through 0.45 μm sterile filter membranes and then kept in the dark and stored at 4 °C. Percent of extract yields (Table 1) were calculated with the following formula:

$$\mathrm{Extract}\;\mathrm{Yield}\;\left(\%\right)=\frac{\mathrm{Weight}\;\mathrm{of}\;\mathrm{extract}\;\mathrm{plants}\;\mathrm{residues}}{\mathrm{Weight}\;\mathrm{of}\;\mathrm{Plant}\;\mathrm{Raw}\;\mathrm{Sample}}\times \;100$$

### 4.2. Bacterial Strains

The strains used in this study to analyze the antibacterial effect of the plant extracts are clinical isolates of *C. difficile* (strains JT127, JT132, JT136, JT343, and JT345) that are part of the collection of the Medical Research Unit in Infectious and Parasitic Diseases at the Pediatric Hospital of the National Medical Center “Siglo XXI.” All strains were cultured in brain heart infusion (BHI) broth (BD Bioxon, Mexico City, Mexico) and incubated at 37 °C in anaerobic conditions (90% N_2_, 5% CO_2,_ and 5% H_2_) for 48 h.

### 4.3. Antibacterial Activity

The antibacterial activity of the extracts was determined by disk diffusion assay. One hundred microliters of bacterial culture were adjusted to 0.5 McFarland turbidity and spread over Müller-Hinton agar (BD Bioxon, Mexico City, Mexico). A 6 mm disk impregnated with 10 μL of an extract was placed on the surface of the medium. As controls, we used DMSO (Sigma–Aldrich, St. Louis, MO, USA) and vancomycin (Sigma–Aldrich, St. Louis, MO, USA; 5 μg per disk). The plates were incubated for 48 h at 37 °C under anaerobic conditions. The experiments were performed in triplicate for each strain, considering inhibition zone diameters greater than 9 mm. To determine the minimum inhibitory concentration (MIC), the tested extracts were diluted in 10% DMSO by serial dilutions of 1:1, 1:10, 1:100, and 1:1000 and analyzed by disk diffusion assay as previously described [27]. The MIC was considered the lowest dilution with a similar effect to the concentrated extract.

### 4.4. Phytochemical Analysis of the Extracts

The extracts were subjected to qualitative phytochemical screening following standard colorimetric methods to determine the presence of terpenoids, steroids, alkaloids, and polyphenols [28,29,30]. First, 5ml of each plant extract was evaporated and resuspended in 5 mL of chloroform, and these were used for Dragendorff, Salkowski, and Liebermann-Burchard tests. To identify alkaloids, the Dragendorff test was performed. 1ml of the extract resuspended in chloroform was placed in filter paper and spread with Dragendorff agent; if the sample turned orange, it was positive for alkaloids. Salkowski test was performed to determine sterol presence. Plant extracts in chloroform were mixed with 1 drop of H_2_SO_4,_ and if the sample turned dark brown, the sample was positive for sterol. Liebermann-Burchard test was performed to identify terpenes. 1 mL of plant extract resuspended in chloroform was mixed with 5 drops of acetic anhydride and 1 drop of concentrate H_2_SO_4_. If sample colored red, then it is positive to terpenes. Folin–Ciocalteu test was performed to identify polyphenols. The ethanolic plant extract was mixed with Folin–Ciocalteu’s reagent (*v*/*v*) and vortex for 5 min. Then, 1.5 volumes of 20% Na_2_CO_3_ were added. If sample turns blue, then it is positive for polyphenols.

### 4.5. Evaluation of the Synergistic Effects among the Extracts

The synergy among the extracts was determined by disk diffusion assay on Müller-Hinton agar as previously described, incorporating 10 μL of a 1:1 mixture of the extracts in each disk. DMSO was added instead of the second extract to control each extract dilution. The synergistic effect was considered when the inhibition ratio was significantly higher than that of each extract alone.

### 4.6. Action Mechanism of the Extracts

As described above, bacteria were grown to the mid-log phase to identify the possible action mechanism of the extracts against *C. difficile*. Then, 100 μL of cell culture for each strain was incubated with 10 μL of each extract or DMSO for 24 h in anaerobic conditions (as previously described). Then, three action mechanisms were analyzed: effects on cell permeability, DNA synthesis, and protein synthesis. After incubation, 1.5 mL of the culture was centrifuged (10,000× *g* for 5 min at room temperature) to determine cell permeability alterations. The supernatants were filtered through a 0.22 μm pore filter. The absorbance of the supernatants was measured at 260 and 280 nm to quantify the DNA and protein content in a Nanodrop ND-1000 spectrophotometer (Thermo Scientific, Waltham, MA, USA). After centrifugation of 1.5 mL of the culture (10,000× *g* for 5 min at room temperature), the pellets were washed twice with PBS and fractionated by alkaline lysis to determine DNA and protein synthesis inhibition. After centrifugation, supernatants corresponding to the cytoplasmic fraction were measured at 260 and 280 nm using a spectrophotometer to quantify total DNA and protein content [31].

As a control, the strains were incubated in the presence of DMSO. If we identified a high quantity of DNA or protein in the supernatant, we hypothesized that the mechanism was induction of membrane permeability; if we identified a lower amount of DNA or protein in the cytoplasm, we assumed that the mechanism was inhibition of macromolecular synthesis inhibition.

### 4.7. Motility Assays

Swarming motility was performed with slight modifications as previously re-ported, [11]. *C. difficile* strains were cultured during 18 h in Brucella under anaerobic conditions at 37 °C. The bacterial number was 0.5 McFarland turbidity (equivalent to approximately 1.4 × 10^7^ CFU/mL based on the Clinical and Laboratory Standards Insti-tute, CLSI) and100 μL of the culture was incubated with 10 μL of each plant extract for 30 min. Afterward, 5 μL was inoculated 0.4% soft BHI agar. After 24 h, swarming mo-tility was determined by measuring the motility ratio. Assays were performed in trip-licate for each strain in independent experiments.

### 4.8. Adherence Assay

The adherence assays of *C. difficile* strains was made on HT-29 cells (ATCC HTB-38). The cells were cultured in DMEM with 25 mM glucose (GIBCO) supple-mented with 10% fetal calf serum (GIBCO) until reaching a confluence of 70%. Contin-uing, the cells were transferred to 96-well plates and cultured to a 90% confluence fol-lowed by three washed with PBS (Merck). Bacteria were grown anaerobically in Bru-cella broth for 48 h at 37 °C (0.5 McFarland turbidity) and 100 μL of the culture was in-cubated for 30 minutes with 10 μL of each plant extract. Then, HT-29 cells were infect-ed with C. difficile strains (10^7^ bacteria/mL) for 1 h under anaerobic conditions at 37 °C and washed four times with PBS to remove nonadherent bacteria. The attached bacte-ria were removed with 100 μL of 0.06% Triton X-100 when incubated by 30 min at 37 °C. Three dilutions (1:10, 1:100 and 1:1000) s were plated onto Casman blood agar and grown anaerobically at 37 °C during 48 h. The percentage of bacteria adhered to the cell monolayers was calculated using the following formula:

$$\mathrm{Adherence}\;\mathrm{Efficiency}\;\left(\%\right)=\frac{\mathrm{Adherent}\;\mathrm{Bacterial}\;\mathrm{Cells}}{\mathrm{Initial}\;\mathrm{Inoculum}+\mathrm{Adherent}\;\mathrm{Bacterial}\;\mathrm{Cells}}\times \;100$$

### 4.9. Sporulation Assays

Sporulation assays were employed to evaluate the influence of the plant extracts on the sporulation rate of the strains. *C. difficile* strains were cultured on Brucella broth at anaerobically conditions and for 48 h at 37 °C. and. In addition, 100 μL of bac-teria (adjusted to 0.5 McFarland) and 10 μL of each plant extract were incubated for 30 minutes. After that, cultures were subjected to alcohol shock with 100 of 80% ethanol for 45 min. Samples diluted in Brucella broth, and 10-fold serial dilutions were cultured on Casman blood agar. Plates were incubated anaerobically for 48 h, and then CFUs were enumerated. The sporulation efficiency was calculated using the following for-mula:

$$\mathrm{Sporulation}\;\mathrm{Efficiency}\;\left(\%\right)=\frac{\mathrm{Sporulated}\;\mathrm{Cells}}{\mathrm{Initial}\;\mathrm{Inoculum}+\mathrm{Sporulated}\;\mathrm{Cells}}\times 100$$

### 4.10. Germination Assays

Germination assays were used to determine the effect of the plant extracts on the strains [32]. *C. difficile* strains were cultured anaerobically on Brucella broth for 48 h at 37 °C, and 100 μL of the bacterial culture (0.5 McFarland turbidity) was subjected to alco-hol shock by mixing the bacteria with 100 μL of 80% ethanol and incubated for 45 min. Samples were diluted in Brucella broth and 10-fold serial dilutions were incubated on-to Casman blood agar for 48 h to calculate the CFUs. The germination efficiency was destimated using the following formula:

$$\mathrm{Germination}\;\mathrm{Efficiency}\;\left(\%\right)=\frac{\mathrm{Germinated}\;\mathrm{Cells}}{\mathrm{Initial}\;\mathrm{Inoculum}+\mathrm{Germinated}\;\mathrm{Cells}}\times \;100\;$$

### 4.11. Statistical Analysis

The data represent the mean of three independent replicates. The results were subjected to one-way ANOVA, and the mean comparisons were performed by Tukey’s post hoc test using ASTATSA software (https://astatsa.com accessed on 27 November 2019) Differences between means were considered significant at a *p*-value < 0.05.

## 5. Conclusions

This study validates the hypothesis that crude ethanol plant extracts can be used against CDI. Our results demonstrate that rosemary, mint, and estafiate extracts significantly inhibit *C. difficile* strains, comparable with vancomycin. Additionally, the action mechanism of each extract varies, suggesting that the mixture of the extracts targets the pathogen via different pathways. All the extracts decreased the motility of bacteria by 40%, but the thyme and ajenjo extracts were the best extracts to block the adherence of bacteria. The rue extract is the best extract to inhibit the sporulation of strains. However, none of the extracts affected germination. Identifying the ideal extracts to block the most significant virulence activities will allow us to obtain phytopharmaceuticals that help fight against them.

## Figures and Tables

**Table 1:** Selected plants used in Mexican traditional medicine against gastrointestinal infections.

Plant Extract	Common Name	Parts Used	Yield (%)	Extract Concentration (mg/mL)	MIC (mg/mL)	Phytochemical Analysis
*Ruta graveolens* (Ru)	Rue	Aerial	8.3	5.01	0.5	Alkaloid, sterol, Terpenoid, and polyphenols
*Rosmarinus officinalis* (Ro)	Rosemary	Leaves	10.4	13	0.13	Alkaloid, sterol, and polyphenols
*Dysphania ambrosioides* (Dy)	Epazote, Mexican Tea, Jesuit’s tea	Leaves	12.3	39.5	3.9	Alkaloid and terpenoid
*Datura ferox* (Da)	Toloache, Long Spined thorn apple, Angel’s trumpets	Leaves	10.7	17.3	0.85	Alkaloid, Terpenoid, and polyphenol
*Mentha piperita* (Me)	Mint	Leaves	7.8	9.3	0.46	Polyphenol
*Thymus vulgaris* (Tm)	Thyme	Leaves	8.2	9.8	0.49	Alkaloid, Terpenoid, and polyphenol
*Artemisia absinthium* (Aj)	Ajenjo, wormwood	Aerial	3	4.8	0.48	Sterol and Terpenoid
*Artemisia ludoviciana* (Es)	Estafiate, Silver, Louisiana wormwood	Leaves	8.1	9.7	0.48	Alkaloid and polyphenol

## Data Availability

Not applicable.
